# Accuracy of Endoscopy in Predicting the Depth of Mucosal Injury Following Caustic Ingestion; a Cross-Sectional Study 

**Published:** 2017-06-22

**Authors:** Athena Alipour-Faz, Maryam Yousefi, Hassan Peyvandi

**Affiliations:** 1 Clinical Research Development Center of Loghman Hakim Hospital, Shahid Beheshti University of Medical Sciences, Tehran, Iran.; 2 Hearing Disorders Research Center, Shahid Beheshti University of Medical Sciences, Tehran, Iran.

**Keywords:** Caustics, endoscopy, gastrointestinal, pathology, data accuracy

## Abstract

**Introduction:**

Esophagogastroduodenoscopy (EGD) is currently considered as the primary method of determining the degree of mucosal injury following caustic ingestion. The present study aimed to evaluate the screening performance characteristics of EGD in predicting the depth of gastrointestinal mucosal injuries following caustic ingestion.

**Methods:**

Adult patients who were referred to emergency department due to ingestion of corrosive materials, over a 7-year period, were enrolled to this diagnostic accuracy study. Sensitivity, specificity, positive and negative predictive values as well as negative and positive likelihood ratios of EGD in predicting the depth of mucosal injury was calculated using pathologic findings as the gold standard.

**Results:**

54 cases with the mean age of 35 ± 11.2 years were enrolled (59.25% male). Primary endoscopic results defined 28 (51.85%) cases as second grade and 26 (48.14%) as third grade of mucosal injury. On the other hand, pathologic findings reported 21 (38.88%) patients as first grade, 14 (25.92%) as second, and 19 patients (35.18%) as third grade. Sensitivity and specificity of endoscopy for determining grade II tissue injury were 50.00 (23.04-76.96) and 47.50 (31.51-63.87), respectively. These measures were 100.00 (82.35-100) and 80.00 (63.06-91.56), respectively for grade III. Accuracy of EGD was 87.03% for grade III and 48.14% for grade II.

**Conclusion:**

Based on the findings of the present study, endoscopic grading of caustic related mucosal injury based on the Zargar’s classification has good accuracy in predicting grade III (87%) and fail accuracy in grade II injuries (48%). It seems that we should be cautious in planning treatment for these patients solely based on endoscopic results.

## Introduction

Ingestion of corrosive substances causes harmful injuries to the upper gastrointestinal tract (1). It is often difficult to estimate the severity of injuries and prognosis of victims based on primary clinical presentation (2). There is a direct correlation between the grade of injury and final outcome (3).

Technetium 99m sucralfate swallow and endoscopic ultrasonography of gastric wall are successfully used in evaluating and predicting the depth of mucosal injuries related to the ingestion of corrosive material (4, 5).

The role of computed tomography (CT) scan in determining the depth of injury and estimating the probability of complication following caustic ingestion has been evaluated in some studies (3, 6, 7). Bahrami-Motlagh and their colleagues declaring the useful role of CT scan as a noninvasive and sensitive screening tool showed the weak correlation of CT and esophagogastroduodenoscopy (EGD) regarding the grading of mucosal injury (7).

EGD is another helpful and available choice in this regard (1, 8). EGD is currently considered as the primary method of determining the degree of injury (1, 8-10). Zargar’s endoscopic classification is recognized as the most famous and acceptable method to predict the prognosis of corrosive ingestion (1). Cheng et al., declared the useful role of EGD in predicting short and long term outcomes as well as treatment planning of these patients (11). The present study aimed to evaluate the screening performance characteristics of EGD in predicting the depth of gastrointestinal mucosal injuries following caustic ingestion.

## Method

***Study design and setting***

This retrospective cross sectional study (diagnostic accuracy study), was conducted on adult patients who were referred to Loghman Hakim Hospital throughout a 7-year period, from 1999 to 2006, due to corrosive material ingestion. This hospital is known as a toxicology referral center for Tehran, Iranian capital. The protocol of this study was approved by the ethics committee of Shahid Beheshti University of Medical Sciences. Researchers adhered to all of Helsinki recommendations and confidentiality of patients’ information.

***Participants***

Adult patients (≥ 15 years) with available data regarding both endoscopic and pathologic grading of mucosal injury following caustic ingestion were enrolled to the study. Patients with obvious surgical indications such as acute peritonitis, pneumo mediastinitis and neck emphysema, who underwent emergent surgical interventions, as well as those with normal or grade I injury on endoscopic findings were excluded. Since grade I mucosal damage could be approached noninvasively and grade IV usually underwent emergent surgical interventions, only patients with grade II and III mucosal injury were enrolled.

***Data gathering***

Patients’ data consisted of baseline variables (age, sex) as well as results of endoscopic and pathologic grading of mucosal injuries, which were collected from patients’ profiles using a predesigned checklist by a senior surgery resident. All endoscopies were performed by expert gastroenterologists within 24 hours of corrosive material ingestion. Tissue biopsies were sent to pathologists who were blind to endoscopic findings in order to report the grading of mucosal damages. Modified Zargar’s endoscopic classification for caustic injuries has been used for endoscopic grading (1).

***Statistical analysis***

Data were analyzed using SPSS version 20. Findings were reported as mean ± standard deviation or frequency and percentage. Screening performance characteristics of EGD endoscopy consisted of sensitivity, specificity, positive and negative predictive values as well as negative and positive likelihood ratios, which were calculated using Med Calc 14 software and reported with 95% confidence interval (CI). Pathologic findings regarding the grade of injuries were considered as the gold standard. Accuracy of 90 - 100 was considered as excellent, 80 – 90 as good, 70 – 80 fair, 60 – 70 poor and < 60 fail. 

## Results

118 patients referred to emergency department following caustic ingestion during the study period. 19 (16.10%) patients underwent emergent laparotomy and 99 (83.89%) underwent EGD endoscopy (45 grade I and 54 grade II and III).

**Table 1:** Screening performance characteristics of endoscopy in grading of mucosal injuries following caustic ingestion with 95% confidence interval (CI)

**Characteristics **	**Grade II (95% CI)**	**Grade III (95% CI)**
**Sensitivity**	50.00 (23.04-76.96)	100.00 (82.35-100)
**Specificity**	47.50 (31.51-63.87)	80.00 (63.06-91.56)
**Positive Predictive Value**	25.00 (10.69-44.87)	73.08 (52.21-88.43)
**Negative Predictive Value**	73.08 (52.2-88.43)	100.00 (87.66-100)
**Positive Likelihood Ratio**	0.95 (0.52-1.74)	5.00 (2.58-9.70)
**Negative Likelihood Ratio**	1.05 (0.57-1.95)	0.00 (0.00-NaN)

**NaN:** the calculation cannot be performed because the values entered include one or more instances of zero.

Finally 54 cases with the mean age of 35 ± 11.2 years were enrolled in the study (59.25% male). Primary endoscopic results defined 28 (51.85%) cases as second grade and 26 (48.14%) as third grade of mucosal injury. On the other hand, pathologic findings reported 21 (38.88%) patients as first grade, 14 (25.92%) as second grade, and 19 patients (35.18%) as third grade of mucosal injury. Table 1 shows the screening performance characteristics of EGD endoscopy for detection of second and third degree mucosal damage following caustic ingestion. Accuracy of EGD endoscopy was 87.03% for grade III and 48.14% for grade II.

## Discussion

Based on the findings of the present study, endoscopic grading of caustic related mucosal injury based on the Zargar’s classification has good accuracy in predicting grade III (87%) and fail accuracy in grade II injuries (48%). It seems that we should be cautious in planning treatment for these patients solely based on endoscopic results. 

There has not been any consensus regarding a standard method in evaluating the severity of injury after ingestion of corrosive material. In spite of current controversies, EGD has been known as the most decisive method for evaluating the mucosal damages (12). EGD is safe concerning the perforation risk 12 to 72 hours post ingestion injury but should be avoided between the 5^th^ and 15^th^ days post ingestion (1, 8, 9). 

However, defining the depth of damage by inspecting the dead epithelium is difficult. There are also some limitations regarding precise diagnosis, especially in first and second grade injuries. Moreover, in the presence of damage to proximal third of the esophagus, endoscopy of distal parts and stomach would be impossible (13). 

In the observational study of Cabral et al. all patients with one week bad outcome had initial severe grading of injury in their emergency endoscopy (14). The sensitivity of endoscopy in predicting the grades 2b and 3 of mucosal damage were 100% and 80% and its specificity were 38% and 37% in the study by Lurie et al. (15). 

Based on the findings of the present study and considering the low sensitivity and specificity of endoscopy in estimation of the depth of grade II tissue injuries, it seems that making a decision regarding a treatment plan for these patients solely based on the results of endoscopic grading is not accurate. In other words, out of the 28 patients that had a grade II tissue injury according to endoscopy results, 21 were placed in grade III after pathology evaluations; therefore, it can be said that relying on endoscopy findings would have led to 75% underestimation of injuries in these patients. On the other hand, only 7 out of the 14 cased that were identified as grade II via pathology had been correctly identified via endoscopy and the other 7 cases were wrongly identified as grade III. This means that about 50% overestimation had occurred in this regard.

However, regarding grade III injuries, endoscopy had a higher accuracy. Considering its 100% sensitivity, it can be considered as a proper screening tool for grade III injuries. In other words, all of the 19 cases that were identified as grade III injuries via pathology had been correctly identified via endoscopy and out of the 26 cases identified as grade III injuries via endoscopy, only 7 (26.9%) were false positive cases.

Based on our findings, we should seek new methods with better diagnostic power to evaluate the depth of injury due to corrosive ingestion. Since third grade injury accompanies clinical signs and symptoms and demands emergent surgical intervention, EGD would be an acceptable diagnostic tool for third grade injuries; whilst in second grade damage, EGD alone is not sufficient to evaluate the depth of damage. Therefore, we recommend other diagnostic tools such as Thechnetium-99 pyrophosphate scintigraphy and endoscopic ultrasonography in order to increase the diagnostic precision of EGD in grade II injuries (16, 17). Finally, our findings show that EGD is not accurate in defining the depth of a mild damage after corrosive agent ingestion, so the treatment approach should not be based on EGD findings; but in severe damages, EGD is accurate in evaluating the depth of damage. Overall, we need accurate and precise tools to evaluate the depth of damage in lower grades of caustic injuries.

*Limitations*

Carrying out the study in a retrospective manner and based on the data available in the profiles was among the most important limitations of this study. In addition, the sample size was determined based on the available cases during a determined period and this might have affected the final power of the results. Therefore, repeating this study in a prospective manner and with a higher number of patients can be helpful.

## Conclusion

Based on the findings of the present study endoscopic grading of caustic related mucosal injury based on the Zargar’s classification has good accuracy in predicting grade III (87%) and fail accuracy in grade II injuries (48%). It seems that we should be cautious in planning treatment for these patients solely based on endoscopic results. 
